# Impact of male trait exaggeration on sex-biased gene expression and genome architecture in a water strider

**DOI:** 10.1186/s12915-021-01021-4

**Published:** 2021-04-30

**Authors:** William Toubiana, David Armisén, Corentin Dechaud, Roberto Arbore, Abderrahman Khila

**Affiliations:** 1 Institut de Génomique Fonctionnelle de Lyon, Université de Lyon, Université Claude Bernard Lyon1, CNRS UMR 5242, Ecole Normale Supérieure de Lyon, 46, allée d’Italie, 69364 Lyon Cedex 07, France; 2 Present address: Department of Ecology and Evolution, University of Lausanne, CH-1015 Lausanne, Switzerland; 3 Present address: Instituto Gulbenkian de Ciência, Rua da Quinta Grande 6, 2780-156 Oeiras, Portugal

**Keywords:** Sexual dimorphism, Sexual selection, Scaling relationships, Sex-biased genes, Genome architecture

## Abstract

**Background:**

Exaggerated secondary sexual traits are widespread in nature and often evolve under strong directional sexual selection. Although heavily studied from both theoretical and empirical viewpoints, we have little understanding of how sexual selection influences sex-biased gene regulation during the development of exaggerated secondary sexual phenotypes, and how these changes are reflected in genomic architecture. This is primarily due to the limited availability of representative genomes and associated tissue and sex transcriptomes to study the development of these traits. Here we present the genome and developmental transcriptomes, focused on the legs, of the water strider *Microvelia longipes*, a species where males exhibit strikingly long third legs compared to females, which they use as weapons.

**Results:**

We generated a high-quality genome assembly with 90% of the sequence captured in 13 scaffolds. The most exaggerated legs in males were particularly enriched in both sex-biased and leg-biased genes, indicating a specific signature of gene expression in association with trait exaggeration. We also found that male-biased genes showed patterns of fast evolution compared to non-biased and female-biased genes, indicative of directional or relaxed purifying selection. By contrast to male-biased genes, female-biased genes that are expressed in the third legs, but not the other legs, are over-represented in the X chromosome compared to the autosomes. An enrichment analysis for sex-biased genes along the chromosomes revealed also that they arrange in large genomic regions or in small clusters of two to four consecutive genes. The number and expression of these enriched regions were often associated with the exaggerated legs of males, suggesting a pattern of common regulation through genomic proximity in association with trait exaggeration.

**Conclusion:**

Our findings indicate how directional sexual selection may drive sex-biased gene expression and genome architecture along the path to trait exaggeration and sexual dimorphism.

**Supplementary Information:**

The online version contains supplementary material available at 10.1186/s12915-021-01021-4.

## Background

Sexual dimorphism, or phenotypic differences between males and females of the same species, is one of the most common sources of phenotypic variation in nature [1, 2]. Understanding how this process is regulated in a sex-specific manner at the genomic level still poses an important challenge [3]. Differences in gene expression have emerged as a common mechanism to explain phenotypic differences among individuals sharing almost the same genome [4, 5]. In the last decade, a large number of studies have characterized genes with sex-biased expression in a variety of species, leading to an emerging framework attempting to link sex-biased gene expression to phenotypic divergence of the sexes [4–6]. Other mechanisms that may explain the evolution of sexual dimorphism have also been documented, including analyses of signature of selection in coding sequence [7, 8]. Elevated rates of sequence evolution, when detected in the set of genes that are sex-biased, are often interpreted as a sign of adaptive evolution caused by sexual selection and, in some cases, the correlation with sexual dimorphism is particularly appealing [9, 10]. The development of whole genome sequencing techniques also made it possible to assess the genomic distribution of genes associated with sexual dimorphism. Recent studies have notably shown that sex-biased or sex-specific genes tend to be unevenly distributed between chromosomes (e.g., X chromosome versus autosomes), sometimes even forming gene clusters within chromosomes, highlighting a possible role of sexual selection in driving genome evolution [11, 12].

Among the countless examples of sexual dimorphism, some species have evolved extreme characters whereby males, generally, develop such drastic phenotypes that they appear exaggerated compared to homologous traits in the other sex or to other body parts [13–16]. These growth-related secondary sexual traits have received considerable attention in developmental genetics, but we still lack a general understanding of the genomic regulation underlying their development [13, 17–27]. In addition, studies of sexual dimorphism tend to focus on adult gonads or whole-body transcriptomic datasets, which are unsuited for understanding how secondary sexual characters are built during development and their possible consequences on genome evolution [4, 5, 28–33]. Conversely, while some studies in flies examined sex-biased gene expression underlying sex differences in bristle patterns [34], most studies across tissues and developmental stages lack comparisons between the sexes [35, 36]. We, therefore, know little about which sets of developmental genes are associated with trait exaggeration, whether they present a pattern of sequence evolution or whether they tend to be arranged in any specific genomic organization. Developmental genes are often known to be highly pleiotropic, which may in turn influence the genomic architecture associated with trait exaggeration due to developmental constraints [37–39].

**Fig. 1:**
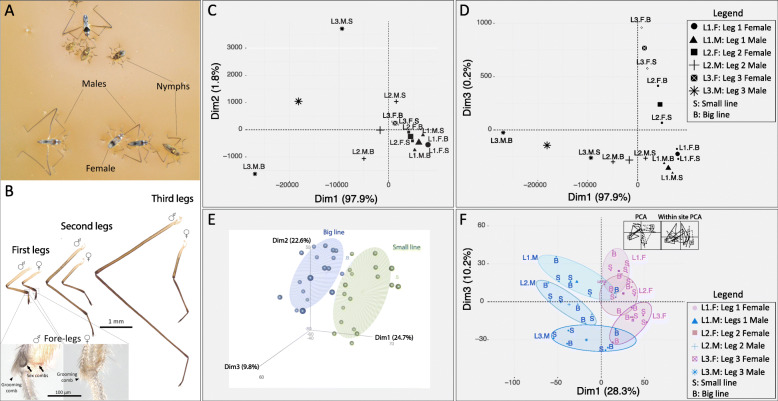
**a** *Microvelia longipes* in the wild. **b** Sexual dimorphism in the legs, showing differences in length and male-specific sex combs in the first-legs (Inset). **c**, **d** Principal component analysis (PCA) on measurements of male and female leg length from the long-leg and short-leg selected inbred populations, also used for the transcriptomic analyses [41]. **c** The first principal component (Dim1) explains primarily differences between legs of the same sex while the second PCA (Dim 2) explains differences between inbred populations, specifically in males. **d** The third PCA (Dim 3) explains the differences between the sexes. **e**, **f** Principal Component Analysis on the whole transcriptomic dataset. **e** The three first PCAs (Dim1, 2, 3) recapitulate the variance between the Big (blue) and Small (green) lines. **f** Within-class analysis after correcting for line effects. Dimension 1 separates sexes while Dimension 3 separates legs. The inset represents the within-class correction for the line effects

We aimed here to assess how ontogenetic sexual dimorphism is associated with sex-specific regulation of gene expression and genome architecture in the water strider *Microvelia longipes* (Heteroptera, Gerromorpha, Veliidae), an emerging model in the field of sexual selection and trait exaggeration [40, 41]. *M. longipes* is a hemimetabolous insect that displays a striking case of male-specific exaggerated trait where some males develop extremely long third legs compared to females (Fig. 1a). The length of the third legs in males is under strong directional sexual selection and these legs are used as weapons to kick opponents away from the sites where females mate and lay eggs [41]. Such directional selection is associated with the evolution of disproportionate growth (i.e., hyperallometry) in male third legs. Here we study the genomic regulation underlying the elaboration of this exaggerated phenotype in order to shed light on the role of sexual dimorphism in shaping genome evolution. We generated a high-quality genome of *M. longipes*, with chromosome-scale resolution, and compared the expression, molecular evolution, and genomic location of sex-biased genes in the three pairs of legs at a developmental stage where the legs diverge between the sexes [40]. Combined, our approach first identified signatures of trait exaggeration in terms of sex-biased gene expression patterns and sequence evolution. Second, it characterized chromosomes and genomic regions that are enriched in sex-biased genes associated with the directional sexual selection applying to male exaggerated legs in *M. longipes*.

## Results

### De novo assembly and automatic annotation of *M. longipes* genome

To study the genetic mechanisms underlying extreme growth of male legs, we generated de novo the genome of *M. longipes* (Fig. 1a) using lines established from a French Guiana population that were inbred through 15 brother-sister crosses [41]. Next-generation sequencing and k-mer frequency distribution in raw sequencing reads estimated *M. longipes* genome size to about 0.67 Gb (see Additional file 1: Table S1 for metrics). Genome assembly combined multiple mate-pair Illumina libraries, PacBio, and Dovetail Hi-C/Hi-Rise libraries [42–44] (Additional file 1: Table S1; see the “Methods” section). The final assembly generated chromosome-length scaffolds with scaffold N50 = 54.15 Mb and contig N50 = 216.72 Kb (Additional file 1: Table S1). Over 90% of *M. longipes* genome is represented in the thirteen largest scaffolds (Additional file 1: Table S1).

We then used automatic genome annotation, supported by de novo transcriptome-based gene models, to build the gene set of *M. longipes* (see the “Methods” section). This analysis predicted 26,130 genes and 27,553 transcripts. BUSCO analysis, based on the 2018 insect dataset [45], revealed that 96% of gene models are present; among these 92% are complete, 3% are fragmented and 1% are duplicated (Additional file 2: Figure S1). We therefore conclude that *M. longipes* genome is near-complete.

### Variation in gene expression explains differences in leg length

In *M. longipes*, the most obvious difference between legs is reflected in their size (Fig. 1a, b), at the exception of a sex comb which is present in males’ first legs but not females’ first legs (Insets in Fig. 1b) [41]. Principal component analysis (PCA) of adult male and female leg length from two isogenic lines (hereafter called big and small lines), selected for differences in absolute leg length [41], revealed that the first major component of variation encompassed 97% of the total variation and separated samples based on pairs of legs in both sexes, with male third legs covering most of the divergence (Fig. 1c). The second and third major axes of variation discriminated males from the two lines and sexes, respectively, although they only contributed 2% to the total variation (Fig. 1c, d).

To test whether these phenotypic differences correlate with variation in gene expression, we sequenced the leg transcriptomes of males and females from these lines at the 5th nymphal instar – the developmental stage where we observed a burst of growth [40]. If a strict correlation existed between leg length and gene expression, we should predict samples to cluster by pairs of legs, then by line, and finally by sex. Instead, the three first major axes of variation in the leg transcriptomes clustered samples based on line (Fig. 1e). The line effect accounted for about 60% of the total variation in gene expression, thus potentially hiding signals associated with differences between legs and sexes. We therefore corrected for this line effect, using within-class analysis, and generated a new PCA that now separates the sexes in PC1 (28.3% of variation in gene expression), and the legs of the same sex in PC3 (10.2% of the variation in gene expression) (Fig. 1f). We conclude that the three main components involved in leg length variation were retrieved in our transcriptome datasets. Yet, conversely to leg morphologies, homologous legs from the two sexes are now more divergent in terms of gene expression than legs from the same sex, consistent with previous findings in flies [34]. We hereafter focus on the effect of sex on gene expression as it potentially represents a major factor underlying leg exaggeration through differences in allometric coefficients [41] (Additional file 3: Figure S2).

### Leg exaggeration and sex-biased gene expression

**Fig. 2:**
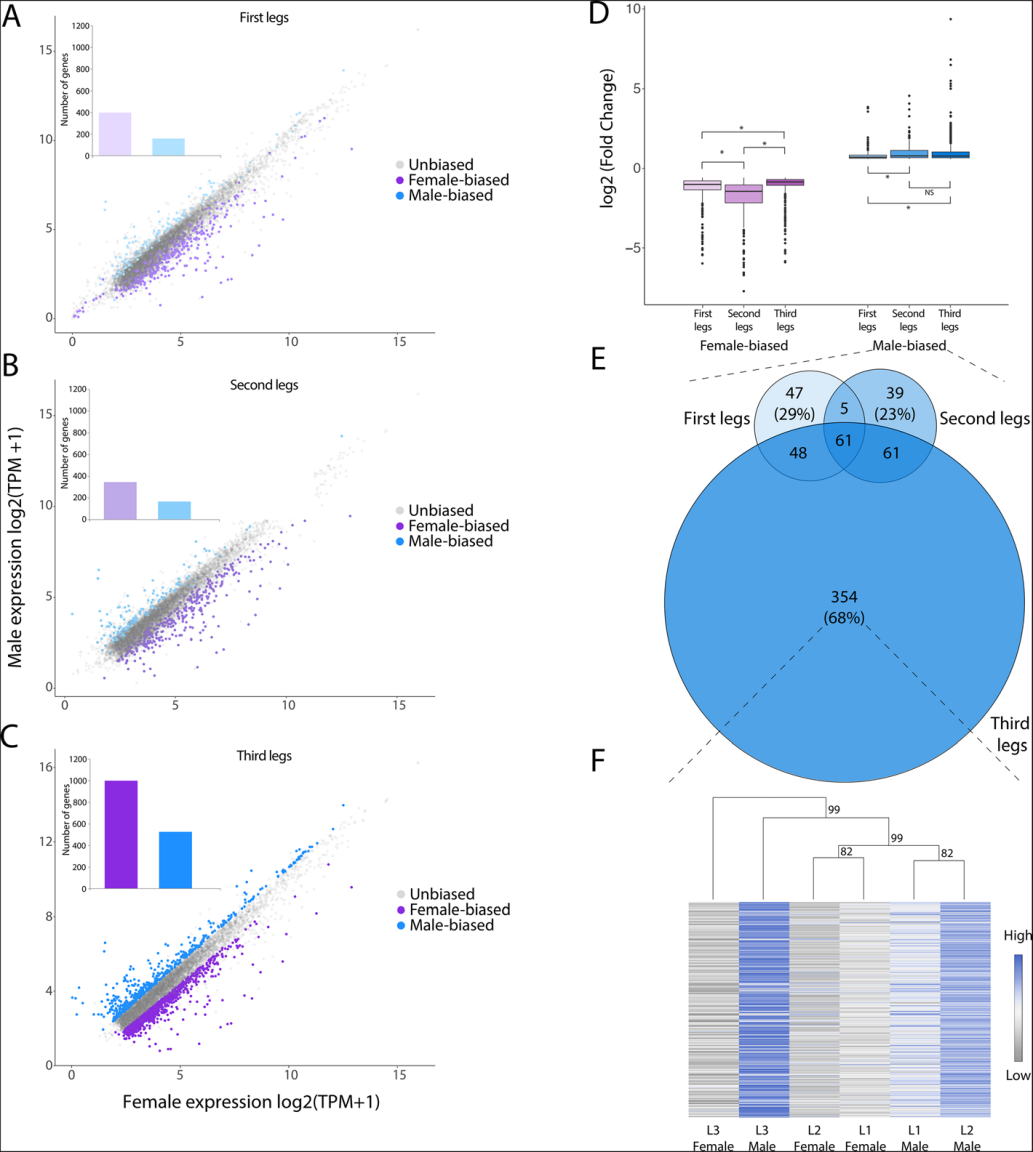
Signature of trait exaggeration among sex-biased genes. **a**–**c** Comparison of gene expression (log2 TPM + 1) in male and female legs. Dots highlighted in purple and blue represent genes with significant difference in expression in females and males respectively. Insets indicate the number of female- and male-biased genes in each leg. **d** Differences in fold change (Wilcoxon tests) among the sex-biased genes identified in the three pairs of legs independently. **e** Venn diagrams of the male-biased genes identified in the three pairs of legs. Size of the diagrams is proportional to the total number of genes. **f** Hierarchical clustering (1000 bootstraps) and heatmap based on average leg expression in males and females for the genes with significant male-biased expression specifically in the third legs of males (*n* = 354)

The legs of *M. longipes* males and females differ in their scaling relationships and degree of exaggeration [41] (Fig. 1a–d, Additional file 3: Figure S2). To determine more specifically the patterns of gene expression underlying the observed sexual dimorphism in scaling relationships, we compared gene expression profiles of homologous legs between the sexes. We found that the legs of *M. longipes* at the fifth nymphal instar consistently expressed about 30% of the genome (Table 1). All three legs showed about twice as many female-biased than male-biased genes, with the third legs having the highest total number of sex-biased genes (Fig. 2a–c; Table 1). Interestingly, average sex-bias in gene expression, as measured by log2 fold change, is significantly higher in the second legs and third-legs of males, which are hyper-allometric, than the first-legs, which are iso-allometric (Fig. 2d; Additional file 4: Table S2). This pattern was consistent with the general over-expression of male-biased genes in the two exaggerated legs, especially the third, compared to the first legs (Additional file 5: Figure S3;Additional file 4: Table S2). This correlation was however absent for female-biased genes (Fig. 2d; Additional file 5: Figure S3). These findings suggest that the extent of sex-biased gene expression correlates with the pattern of leg growth in males but not in females. More than two thirds of the male-biased genes in the first and second legs were shared with the most exaggerated legs, whereas about two thirds of male-biased genes in the third leg (*N* = 354) were not shared with the other legs (Fig. 2e). A hierarchical clustering analysis separated males’ third legs from the other legs in both sexes, confirming that on average the sexual dimorphic expression of these 354 genes is restricted to the most exaggerated leg (Fig. 2f). Furthermore, the third legs showed a high number of female-biased genes, despite the lack of exaggeration, suggesting that the development of this extreme sexual dimorphism may also result from the active regulation of specific genes in female’s third legs or their active repression in male’s third legs. Altogether, these results show that the developing third legs display unique patterns of sex-biased gene expression, in terms of number and/or levels of expression, compared to the two other legs. This suggests that heightened sexual dimorphism of *M. longipes* third legs is associated with both increased degree of male-biased expression and the recruitment of new sex-biased genes.

**Table 1:** Number and percentage of genes expressed in the legs in males and females

	Total expressed genes	Male-biased	Female-biased
Leg 1	8950	161 (1.80%)	398 (4.45%)
Leg 2	8706	166 (1.91%)	346 (3.97%)
Leg 3	8710	524 (6.02%)	999 (11.47%)

### Male exaggerated legs are enriched in both leg- and sex-biased genes

**Fig. 3:**
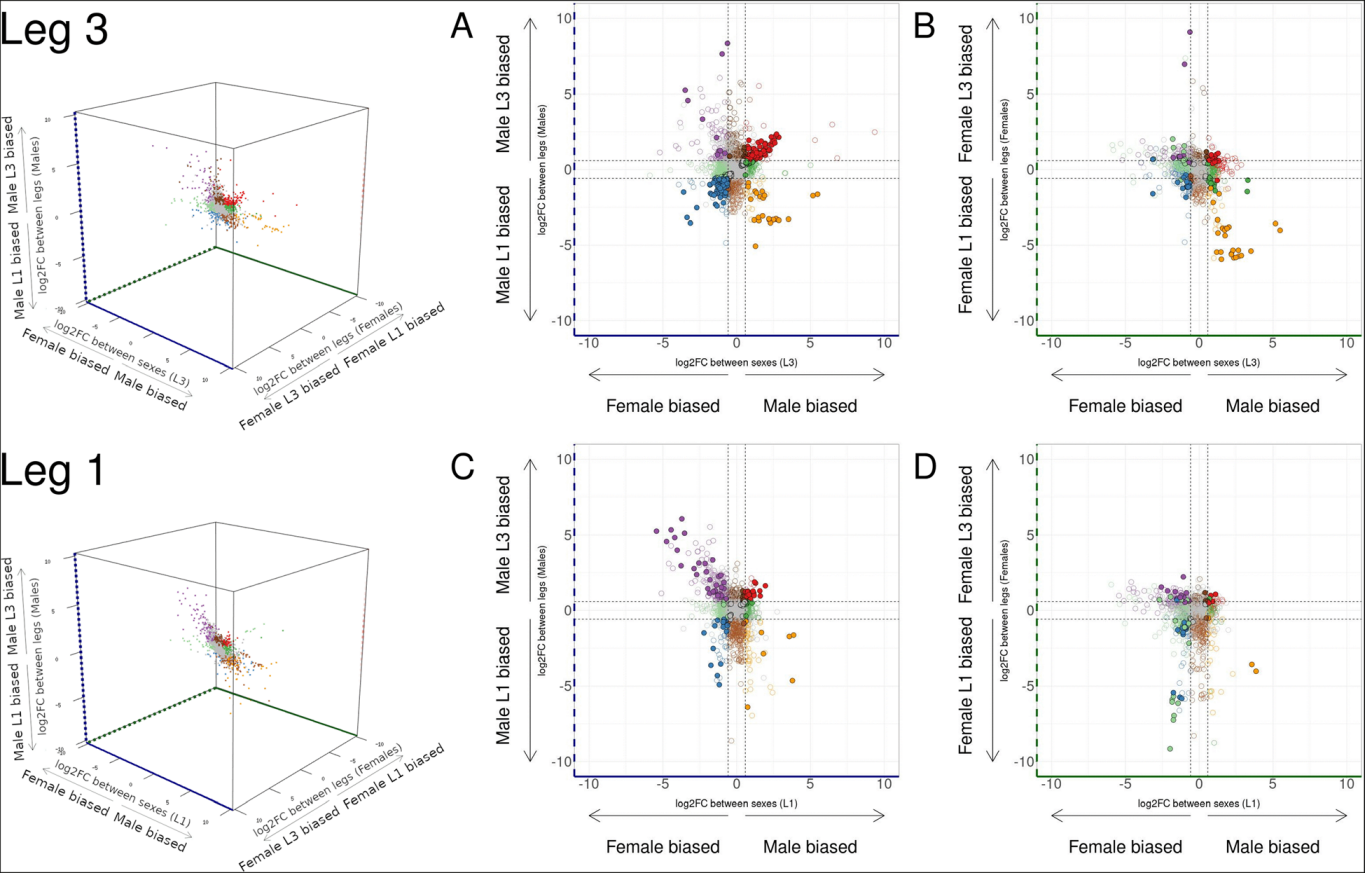
Crosstalk between leg- and sex-biased genes. **a** Comparison between sex-biased genes in the third legs and leg-biased genes in males. **b** Comparison between sex-biased genes in the third legs and leg-biased genes in females. **c** Comparison between sex-biased genes in the first legs and leg-biased genes in males. **d** Comparison between sex-biased genes in the first legs and leg-biased genes in females. Color code in **a** and **b** represents the same genes in these two panels, and color code in **c** and **d** represents the same genes in these two panels. Gens are based on sex-biased and leg-biased expression in males (log2FC > log2(1.5)): purple = female-biased and leg 3 biased; dark brown = sex unbiased and leg 3 biased; red = male-biased and leg 3 biased; light green = female-biased and leg unbiased; gray = sex unbiased and leg unbiased; dark green = male-biased and leg unbiased; blue = female-biased and leg 1 biased; light brown = sex unbiased and leg 1 biased; orange = male-biased and leg 1 biased. Filled circles indicate genes with padj < 0.05 in both conditions (sex- and leg-biased). Hollow circles indicate genes with padj > 0.05 in one or both conditions

Pleiotropy is thought to constrain the evolution of sex-biased gene expression [46], and sexual dimorphism may result from post-transcriptional regulation, possibly resulting in rather broad expression of sex-biased genes [47]. To test the impact of trait exaggeration on gene expression during leg development, we combined our list of leg-biased genes (genes differentially expressed between the first-legs and the third-legs of the same sex) with the list of sex-biased genes (genes differentially expressed between the same leg of males and females), and performed a comparison of fold-change (Fig. 3). Interestingly, we observed that male-biased genes in the third legs tend to be upregulated in the third compared to the first legs of males (84 out of 524 (16.03%); Fisher’s exact test; *p* value 3.48e−31) (red dots in Fig. 3a, Additional file 6: Table S3). Similarly, 80 out of the 856 (9.34%) female-biased genes in the third legs are also upregulated in the first legs compared to the third legs of males (Fisher’s exact test; *p* value 1.62e−14) (blue dots in Fig. 3a, Additional file 6: Table S3). This suggests that male exaggerated legs are enriched in sex- and leg-biased genes.

We therefore compared, in a second step, the association between sex-biased genes in the first legs and their leg-biased expression. Likewise, these associations were dampened in terms of number of genes when we looked at sex-biased genes in the first legs (Fig. 3d, e). Male-biased genes in the first legs were not over-represented among upregulated genes in male first legs (Fisher’s exact test; *p* value 0.61) (orange dots in Fig. 3c). We obtained similar results when we selected leg-biased genes in females with male-biased genes showing no tendency toward leg-biased expression (Fisher’s exact tests; *p* values 0.06 and 0.91) (Fig. 3e, Additional file 6: Table S3). In contrast, female-biased genes in the first legs tend to be differentially expressed between legs, although such association is observed to a lower degree than in the exaggerated third legs (Fig. 3c, d, Additional file 6: Table S3).

In the second legs, which are mildly exaggerated in males, we also recovered a similar pattern of sex and leg-biased gene expression, although with fewer genes as for female-biased genes in the first legs (Additional file 7: Figure S4; Additional file 6: Table S3). Overall, we found an enrichment of genes with both leg- and sex-biased expression that was particularly higher in male exaggerated third legs. This crosstalk highlights possible modularity by which sex-biased genes may have acquired leg-biased expression (or vice versa) in association with the exaggerated growth of male third legs without affecting other organs.

### Sequence evolution of sex-biased genes

**Fig. 4:**
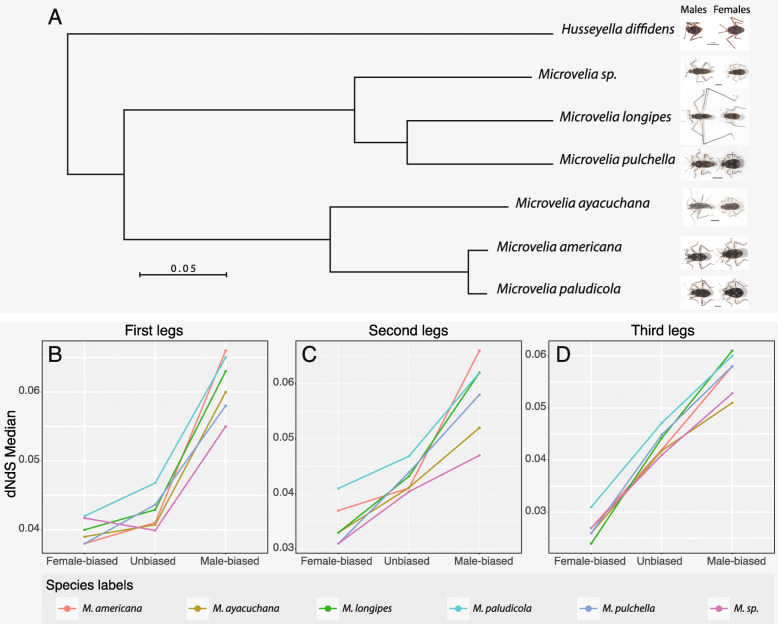
Phylogenetic relationships and sequence evolution across a sample of six *Microvelia* species: *M. longipes*, *M. pulchella, M. ayacuchana*, *M. americana*, *M. paludicola*, and *M. sp* (Cayenne). **a** Phylogeny of Microvelia genus based on 1500 genes and males and females pictures showing leg length exaggeration evolution in *M. longipes*. **b**–**d** Estimation of sequence evolution, using dN/dS of the genes that are male-biased, female-biased or unbiased in the first legs (**b**), second legs (**c**), or the third legs (**d**). Statistical analyses are shown in Additional file 8: Table S4

We have shown that the pattern of sex-biased gene expression in *M. longipes* legs correlated in several aspects with the elaboration of the exaggerated third legs in males. In several species, sex-biased genes display a higher rate of evolution compared to unbiased genes but relatively little is known about their sequence evolution in the context of trait exaggeration [48]. We classified genes in *M. longipes* based on their sex-biased expression pattern in all three legs (male-biased, female-biased and unbiased, respectively) and compared their sequence evolution between each other but also with five additional *Microvelia* species (Fig. 4a–d; Additional file 8: Table S4; Additional file 9: Figure S5; see the “Methods” section). In *M. longipes*, we found that the genes that are male biased in all three legs evolved faster than unbiased and female-biased genes (Fig. 4b–d). However, the pattern of sequence evolution was different for female-biased genes. While the pattern of sequence evolution for female-biased genes in the first legs was similar to that of unbiased genes (Fig. 4b, c, Additional file 8: Table S4; Additional file 9: Figure S5), female-biased genes in the second and third legs evolved slower than unbiased genes (Fig. 4c, d; Additional file 8: Table S4; Additional file 9: Figure S5). These results suggest that the evolution of trait exaggeration was associated with positive selection for male-biased and purifying selection for female-biased genes (Additional file 8: Table S4). Given the association previously observed between sex- and leg-biased expressions, we further classified sex-biased genes into leg-biased and leg-unbiased categories (Additional file 9: Figure S5). Interestingly, we found that the relatively fast evolution of male-biased genes results primarily from genes with both sex- and leg-biased expressions (Additional file 8: Table S4; Additional file 9: Figure S5). This pattern was again not found in female-biased genes as they have similar sequence evolution regardless of their leg-biased expression (Fig. 4d; Additional file 8: Table S4; Additional file 9: Figure S5). When we analyzed sequence evolution of this same set of genes (i.e., sex-biased and leg-biased genes in *M. longipes*) in the remaining five *Microvelia* species, we found that the pattern observed in *M. longipes* was similar across all *Microvelia* species, even though trait exaggeration occurs only in *M. longipes* (Fig. 4b–d; Additional file 8: Table S4; Additional file 9: Figure S5). This result suggests that the increased rate of sequence evolution in this sample of genes preceded the evolution of exaggerated male leg length, and that a set of genes already under sexual selection may have been co-opted during the evolution of exaggerated leg length in *M. longipes*. Determining whether these genes are sex-biased in the other *Microvelia* species will further improve our understanding of the link between sex-specific regulation of gene expression and the evolution of sexual dimorphism.

### Sex-biased gene expression and genome architecture

**Fig. 5:**
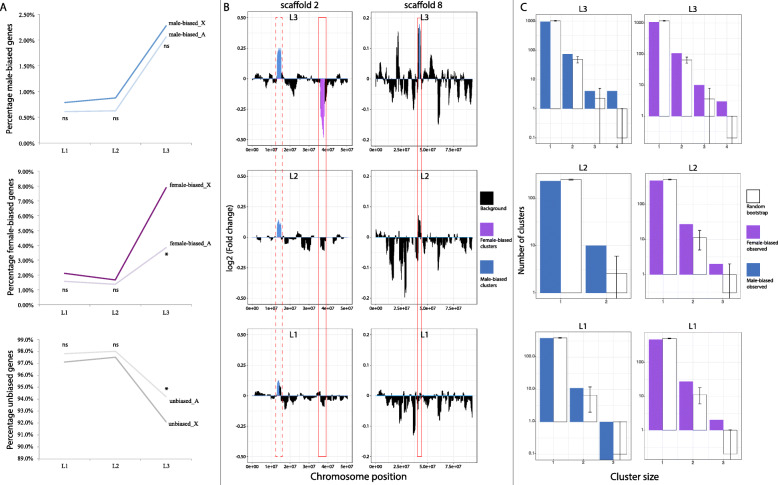
Genomic distribution of sex-biased genes in *M. longipes*. **a** Percentage of male-biased, female-biased and unbiased genes (from top to bottom) in the X chromosome and the autosomes across the three pairs of legs. Biased-distribution of the sex-biased genes on the X chromosome was estimated using Fisher’s exact tests: *p value< 0.05. **b** Large genomic clusters of sex-biased genes along scaffold #2 and the scaffold #8. Clusters highlighted in blue represent genomic regions enriched in male-biased genes. Clusters highlighted in purple represent genomic regions enriched in female-biased genes. Solid red frame indicates genomic clusters enriched in male- and female-biased genes specifically in the third legs. Dotted red frame indicates genomic clusters enriched in male-biased genes in all three legs but with different degrees of fold-change, recapitulating the degree of leg length exaggeration. **c** Genomic clusters of consecutive male- (blue) or female-biased (purple) genes in the three pairs of legs. Cluster size indicates the number of consecutive genes. Note that the y axis is log scaled. Error bars contain 95% of randomly generated bootstrap values

Theory predicts that sexual selection can be an important driver of genome evolution [49, 50], and we sought to test this prediction by analyzing the distribution of sex-biased genes along the genome of *M. longipes.* First, we identified the scaffold that corresponds to the X chromosome (see material & methods). Interestingly, our analysis detected enrichment in the X chromosome with female-biased genes of the third legs, but not the two other legs, compared to the autosomes (Fig. 5a). The proportion of female-biased genes between the X chromosome and the autosomes in the different legs confirmed that the enrichment observed was caused by an accumulation on the X chromosome of genes specifically biased in the third legs of females (Fig. 5a). In contrast, we did not find any significant under- or over-representation of male-biased genes from any of the three legs on the X chromosome (Fig. 5a). Because of the known effect of dosage compensation on the expression of genes located on the X chromosome [4, 51–53], we compared the levels of expression of all X chromosome genes between the sexes. This analysis failed to detect any significant global difference in expression of these genes between males and females, regardless of the legs (Additional file 10: Figure S6), suggesting that dosage compensation occurs in all leg tissues in *M. longipes*.

Another potential effect of sexual selection on genome architecture is through rearrangements of genes or large genomic regions within chromosomes [11, 54–58]. We therefore performed a fine-scale visualization of the sex-biased genes along the thirteen largest scaffolds covering *M. longipes* genome (Fig. 5b; Additional file 11: Figure S7). We recovered few large genomic regions of 2 Mb significantly enriched in sex-biased genes (Additional file 11: Figure S7; see the “Methods” section). These include a total of 100 sex-biased genes (about 2% of the total number of sex-biased genes in all three legs), indicating that only a fraction of sex-biased genes arranges in such large genomic regions (Fig. 5b; Additional file 11: Figure S7). Among these, three large enriched regions located on scaffolds #2 and #8 contained a total of 36 sex-biased genes in the third legs (11 female-biased and 25 male-biased) (Fig. 5b). Interestingly, two of these regions were specific to the third leg whereas the third indicated an enrichment of male-biased genes that were common to the three pairs of legs but with a higher degree of differential expression in the third legs (boxes with solid line and box with dashed line respectively in Fig. 5b). In these regions, we could notably identify several unknown genes (10 out of 36 genes) including a cluster of four that were all strongly male-biased. Protein motif prediction, using Pfam, revealed a conserved domain of several transmembrane motifs in these four protein-coding genes.

Finally, we looked for small clusters of consecutive genes with similar patterns of expression in an attempt to assess common regulation [59]. We found that over 15% of male-biased and over 20% of female-biased genes arranged in clusters of two to four genes in the third legs, while only about 8.5% and 10% respectively are expected under a null hypothesis of random gene order (permutation test: *p* value < 0.05; see material and method) (Fig. 5c; Additional file 12: Figure S8). Specifically, we found up to seven clusters of four consecutive sex-biased genes in the third legs while only a maximum of two of them were expected by random permutation (Fig. 5c). In the second pair of legs, we also found that about 10% of the male- and female-biased genes are arranged in clusters of at least two genes, while 2 to 3% were expected by random permutation (*p* value < 0.05; Fig. 5c; Additional file 12: Figure S8). In comparison with the third legs, clusters of male- and female-biased genes did not exceed two and three consecutive genes, respectively (Fig. 5c). Male-biased genes in the first legs did not show any enrichment in clusters, and only one such cluster of three genes was detected (*p* value > 0.05; Fig. 5c; Additional file 12: Figure S8). However, we found an enrichment of female-biased gene clusters, including 2 clusters of 3 consecutive genes (Fig. 5c; *p* value < 0.05; Additional file 12: Figure S8).

### Molecular function of sex-biased genes

Finally, we aimed to determine the molecular function of the sex-biased genes in our dataset. Gene ontology (GO) term analyses revealed enrichment in translation, metabolic processes, and Wnt signaling pathways for the male-biased genes in the third legs (Additional file 13: Table S5). The “translation” GO term uncovered enrichment for several ribosomal proteins also known to play an essential role in cell proliferation in response to ribosomal stress [60]. We also identified enrichment in molecular functions such as transferase activity indicative of possible post-transcriptional regulation differences between the two sexes. Female-biased genes in the third legs were enriched in various functions such as transcription factor, kinase, or GTPase activities that are probably involved in regulating biological processes such as transcription, metabolism, or signal transduction (Additional file 13: Table S5).

## Discussion

Uncovering the genetic and genomic changes underlying phenotypic divergence between males and females is central to our understanding of phenotypic evolution [5, 14, 49, 61]. As an emerging model, *Microvelia longipes* offers exciting life history and ease of experimental manipulation to study how sexual selection can drive extreme phenotypic divergence between the sexes [40, 41, 62]. The genome sequencing and assembly add a significant resource that will benefit the community in addressing fundamental questions in relation to the genetic and genomic processes underlying, not only the divergence of the sexes, but also phenotypic plasticity within the same sex. In a previous study, we established that the evolution of male third leg exaggeration was associated with intense competition between conspecific males to dominate egg-laying sites [41]. The current study sheds light on the regulatory processes, both developmental and genomic, underlying this male-specific trait exaggeration.

### Trait exaggeration and sex-biased gene expression

Comparing the three pairs of legs in *M. longipes* offers a unique opportunity to understand how gene regulation correlates with phenotypic differences between males and females, as these legs present different types and degrees of sexual dimorphism, including discrete and quantitative phenotypes [40, 41]. For example, the development of the sex-comb, a discrete phenotype known to be under stabilizing selection [63, 64], occurs in the first legs of males during the 5th instar [65]. In contrast, the exaggerated growth of male third legs, a quantitative trait which also occurs during the 5th nymphal instar [40], is an example of directional sexual selection that is absent, or at least reduced, in the two other male legs and in females legs [41]. It is often hypothesized that sex-biased gene expression is correlated with phenotypic dimorphism. This would imply that the genes evolving under strong sexual selection in males for leg exaggeration should be male-biased in expression during the developmental window where divergence in growth rate occurs between the sexes. While our data support this hypothesis, we also found an even larger number of female-biased genes in the third legs (Fig. 2). This is in contrast to a previous study in flies where Barmina et al. compared the developmental transcriptome of first and second legs just before the elaboration of male sex-combs [34]. In contrast to the high number of sex-biased genes we observe in *M. longipes* legs, this fly study only identified a handful in the first legs and none in the second legs [34]. The large differences in the number sex-biased genes in the developing legs between *Microvelia* and *Drosophila* could be explained by the differences in the nature of dimorphism (whether it involves complex or discrete traits) along with the fundamental difference in development mode (holometabolous versus hemimetabolous).

Our data, although representing a snapshot of a specific developmental window across legs and sexes, point to a unique set of genes that are active in the exaggerated leg when compared to its non-exaggerated serial homolog in males or to its homolog in females. This suggests that trait exaggeration is associated with gene regulation in a leg-dependent manner. Genes with female-biased expression may therefore have evolved as a consequence of the strong sexual selection on male exaggerated legs, in addition to the antagonistic selection on females to escape costs associated with superfluous leg elongation. This highlights the need for further studies to understand the regulatory process underlying the development and evolution of various types of secondary sexual traits (e.g., discrete versus quantitative) across distinct modes of development in a comparative framework.

### Trait exaggeration and sequence evolution

It is well established that sex-biased genes tend to show a different rate of sequence evolution compared to unbiased genes, which often indicates signs of adaptive evolution [66, 67]. However, how selection on exaggerated traits manifests itself in the sequence of genes associated with the sex-restricted development of these traits and throughout the evolution of a lineage remains to be tested. In this context, our data show several important insights. First, male-biased genes in *M. longipes* evolved faster than unbiased genes, whereas by contrast, female-biased genes evolved slower than unbiased genes. Second, this increased rate of sequence evolution in male-biased genes is largely driven by the set of genes whose expression profiles are tightly associated with trait exaggeration. Third, the pattern of sequence evolution seen in *M. longipes* is largely shared by five additional *Microvelia* species that do not exhibit any exaggerated leg length. These findings suggest that male-biased genes associated with trait exaggeration are under positive selection, probably due to their reduced pleiotropic effect, also consistent with their expression being enriched in the exaggerated leg. This might free these sequences from developmental constraints and allow for adaptive evolution driven by male competition [41, 67]. Female-biased genes, however, show a clear pattern of purifying selection suggesting that these genes may be involved in various developmental processes thus constraining their evolution. One possible explanation is that these genes are driven by sexual conflict in females favoring much shorter legs as an optimum.

The finding that the genes associated with trait exaggeration in *M. longipes* males show the same pattern of sequence evolution across all other *Microvelia* species is surprising given the clear divergence in the degree and nature of sexual dimorphism across this sample. Although only *M. longipes* exhibits such dramatic dimorphism in leg length, other species present various sexually dimorphic phenotypes ranging from male fighting behavior to the presence of spines and slightly longer legs in males [41]. While we do not have any information about sex-biased expression of these genes in the five additional species, the consistency of sequence evolution between the sexes in this sample suggests that these genes may have already been involved in sexual dimorphism ancestrally in this lineage, and that a subset of them continued to be co-opted for further divergence between the sexes.

### Trait exaggeration and genome architecture

The expression profiles of the active transcripts across *M. longipes* legs represent a valuable resource to inform about the distribution of sex-biased genes in the genome. The X chromosome, for example, has been hypothesized to be a genomic hotspot for sexual selection where female beneficial dominant mutations and male beneficial recessive mutations are expected to accumulate [49, 51, 52]. However, interpreting the representation of sex-biased genes on the X chromosome is often influenced by dosage compensation [4, 51, 52]. In *Drosophila*, for example, the scarcity of male-biased genes on the X chromosome was suggested to result, at least partially, from dosage compensation. Our analyses uncovered a significant enrichment of the X chromosome with female-biased genes in the third legs, but not the two other legs. We also detected that dosage compensation operates in all legs of *M. longipes*, suggesting that this mechanism is unlikely to be responsible for the enrichment observed. Therefore, it is possible that this “feminized” X chromosome represents a mechanism for the resolution of sexual conflict during the evolution of extreme sexual dimorphism in *M. longipes* third legs.

Previous studies have reported large genomic regions and profound genomic rearrangements (e.g. large chromosome inversions) in association with sexually dimorphic characters [11, 55–58]. In contrast, we found relatively few large but many small clusters of sex-biased genes in *M. longipes* genome (Fig. 5). Moreover, these small and large enriched regions seem to be associated with the extreme elongation of male third legs, in terms of number, specificity or degree of differential expression, and may highlight some important genes and regulatory processes involved in sex-specific trait exaggeration. These enriched regions may result, for example, from adaptive gene rearrangement due to shared regulatory elements. Alternatively, they may be a non-adaptive consequence of chromatin-level regulation that prevents some genes from being inactive [59]. Further work, by testing the function of these clustered genes or by comparing the genome architecture of different *Microvelia* species will therefore be necessary to conclude on the adaptive significance of these enriched regions. It is also important to note that the large genomic regions and genomic rearrangements of sex-biased genes reported in previous studies were primarily conducted on primary sexual organs, such as ovaries and testes [11, 54–58]. These tissues are highly complex, often express more sex-biased genes than secondary sexual traits and their evolution is considered to be under natural selection [1, 68–71]. Moreover, analyzing gene expression in these adult tissues does not capture the sex differences that are established during development. In the case of ontogenetic sexual dimorphism, it is expected that sexual selection will act on developmental regulatory processes [5, 17, 72, 73]. In this regard, our results offer the opportunity to test more accurately the role of sexual selection on gene and genome evolution by directly linking the development of sexual dimorphism with patterns and genomic locations of sex-biased genes in the three pairs of legs.

## Conclusions

We identified a signature of leg exaggeration among sex-biased genes. Consistent with studies of sex-biased gene expression [5, 68, 74, 75], we found that the degree of sexual dimorphism in leg length is consistent with different patterns of expression among these genes. In our dataset, the most exaggerated legs mobilized more differentially expressed genes between the sexes and a higher degree of differential expression, especially in male-biased genes. Male-biased genes were significantly fast-evolving whereas female-biased genes were significantly slow evolving compared to unbiased genes. Interestingly, the same genes showed a highly similar pattern of sequence evolution in a sample of five additional species. We found that a large proportion of sex-biased genes, especially in the third legs, displayed also tissue-biased expression (Fig. 3a). Along with other studies showing less pleiotropy for sex-biased than unbiased genes [76, 77], our results point to modularity as a possible mechanism whereby tissues can evolve biased expression freely and acquire sex-specific phenotypes with little deleterious effects. Overall, our findings indicate how directional sexual selection may drive sex-biased gene expression and genome architecture along the path to trait exaggeration and sexual dimorphism.

## Methods

### Population sampling and culture

A *Microvelia longipes* population was collected during fieldwork in French Guyana in Crique Patate near Cayenne in March 2013 [78], and inbred lines were generated from this initial natural population. Two couples were isolates each consisting of one female with a large or a small male respectively. The males were selected based on their absolute third leg size. The crosses were repeated using the progeny of these two initial crosses for the next 15 generations, with a large brother mated with a sister for the big line and a small brother mated with a sister for the small line. After 15 generations of these sibling–sibling inbreeding, the lines were amplified over two generations before phenotyping. The bugs were maintained in the laboratory at 25 °C and 50% humidity in water tanks and fed on crickets.

### Statistics and leg measurements

All statistical analyses were performed in RStudio 0.99.486. For the PCA analysis on leg length, we used twenty males and females from each inbred population and measured them with a SteREO Discovery V12 (Zeiss) using the Zen software.

### Sample collection, assembly, and annotation of the *M. longipes* genome

Hundreds of individuals (males and females mixed) were collected from three inbred populations and frozen in liquid nitrogen before DNA extraction. Genomic DNA was extracted and purified using the Genomic-tip 20/G DNA extraction kit from Qiagen. Genome sequencing, using a mix of Illumina mate pairs and PacBio libraries, was performed at the Beijing Genomics Institute. Chromosome-length scaffold assembly was performed by Dovetail Genomics using Hi-C/Hi-Rise libraries. Additional file 1: Table S1 summarizes the sequencing strategy employed.

The genome sequence was polished using Illumina libraries (Additional file 1: Table S1) and Pilon [79]. Three different automatic annotation strategies, namely Braker, Maker, and StringTie were tested to annotate the genome [80–82]. These annotations were based on the leg transcriptomic dataset generated in this study (36 samples in total), a transcriptome from whole-body individuals collected at all developmental stages (1 sample), and a transcriptome from a third inbred population not mentioned in this study (18 samples). Braker and Maker pipelines also performed de novo automatic annotations.

Maker and Stringtie annotations yielded lower BUSCO quality and manual quality assessment using JBrowse revealed a relatively high number of gene fragmentations that were poorly supported by the alignments. We therefore used Braker annotation for further analyses (Additional file 2: Figure S1).

For Braker annotation, we used Hisat2 alignment files from each transcriptomic sample to train Augustus with UTR option. Final annotation includes 26,130 genes and 27,553 transcripts.

### Sample collection and preparation RNA-sequencing

We collected leg tissues from male and female 5th nymphal instars (2 days after molting within a 6-h time window) that belonged to two inbred populations that differ in average size (see [41]). All individuals were raised in the same laboratory condition and fed with nine fresh crickets every day until the 5th instar. Individuals from the same inbred population were raised in the same water tank. The three replicates of each condition (lines, sexes, and legs) correspond to a pool of 20 individuals chosen randomly (Additional file 3: Figure S2). The dissection of the three pairs of legs, dissociated from the thorax, was performed in RNAlater (Sigma) using fine needles; each pair of legs was incubated immediately on ice in tubes filled with TRIzol (Invitrogen). RNA extractions were performed according to manufacturer protocol. The concentrations were assessed using the Qubit 2.0 Fluorometer (Invitrogen). Quality of RNA samples, library construction, and sequencing were performed by Beijing Genomics Institute. The samples were sequenced using HiseqXten sequencing technology with a paired-end read length of 150 bp.

### Transcriptome assembly, mapping and normalization

Read quality was assessed with FASTQC version 0.10.1 (http://www.bioinformatics.babraham.ac.uk/projects/download.html), and trimmed with TRIMMO-MATIC version 0.32. Specifically, reads were trimmed if the sliding window average Phred score over four bases was < 15, and only reads with a minimum length of 36 bp were kept. Braker annotation was used as a reference for read alignment and the transcriptome quantification. We obtained around 90% alignment rate on the genome and about 72% of uniquely mapped reads using the Hisat2 method (Additional file 14: Table S6). The latter condition was used for the estimation of transcript abundances and the creation of count tables (raw counts, FPKM and TPM tables) were performed using the StringTie pipeline (Additional file 15: Table S7) [82, 83]. The abundance of reads per gene was finally calculated by adding the read counts of each predicted transcript isoforms.

### Comparative transcriptomics: analyses of variance

Initially, the transcriptomic approach was performed on three levels of comparisons; namely the lines, the sexes, and the legs (Additional file 3: Figure S2). The first three axes of variation in gene expression explained 57.1% of the total variation and separated the two inbred populations (Fig. 1e). This confirms the genetic similarity that exists between individuals of the same inbred population. In order to correctly assess the influence of sex and leg comparisons on gene expression variance, we corrected for the line effect using a within-class analysis [84]. Within-class analysis is a method that has been developed for microarray experiments including various factors structuring the data. The objective is to explore the effect of some factors in a multivariate analysis while controlling for several sources of variation from other factors. After correction, the first major axis of variation separated male and female conditions, while PC3 explained the variation between legs (Fig. 1f).

### Identification of sex-biased genes

We first filtered transcripts for which expression was lower than 2 FPKM in more than half of the samples after combining the two inbred populations (12 samples in total). Transcripts with average expression that was lower than 2 FPKM in both males and females were also discarded. The number of reads per “gene” was used to quantify differences in expression among the different conditions of interest using DESeq2 [85]. DESeq2 script is available from Dryad Digital Repository link below and was implemented from the DESeq2 vignette: https://datadryad.org/stash/share/TieHWUYVZWsPHBeCkDkbQ-b_0B3NAsAFy6SUkD4ybrs.

Differential expression analyses between males and females were performed on the two lines combined as we aimed to identify genes involved in male third leg exaggeration, which is a common feature to both lines. The differential expression analysis was also corrected for the line effect and we called sex-biased any gene with a fold-change > 1.5 and a Padj < 0.05. We also ran the differential analysis on lines separately and observed a large overlap with only 20% of sex-biased genes from the two lines separately not included in the combined set (Additional file 16: Figure S9).

### Interaction between leg and sex regulations

In order to detect a possible interaction between leg and sex regulations, we combined our list of sex-biased genes with another list of genes that were identified as differentially expressed between legs of the same sex (i.e., leg-biased genes). Using Fisher’s exact tests, we then identified possible enrichment of genes with both sex- and leg-biased expression among the genes expressed within each tissue.

### Hierarchical clustering

Average expressions of sex-biased genes in the different tissues were clustered using Euclidean clustering in the R package PVCLUST version 1.3-2 [86] with 1000 bootstrap resampling. Heatmaps and clustering were performed using the log2(TPM) average expression of each gene from each tissue. Heatmaps were generated using the R package GPLOTS version 3.0.1.1.

### Estimation of sequence evolution using dN/dS

Whole transcriptomes of five *Microvelia* species (*M. sp*. (Cayenne), *M. pulchella*, *M. paludicola*, *M. Ayacuchana*, and *M. americana*) were sequenced and assembled as in [75]. Additionally, gene sequences for *Microvelia longipes* were extracted from genome-based transcriptome assembly (see the above section). Protein sequences for all transcripts in these *Microvelia* species were retrieved and BLASTP results were used to construct Reciprocal Best Hits (RBH) clusters that include reciprocal best hits between *Microvelia longipes*, *Microvelia pulchella*, and at least a third *Microvelia* species. To improve the accuracy of the alignments we applied a simple length ratio cut-off to assign RBH of 0.5. This filter prevents short transcripts generated by Trinity containing a very well conserved motif to be assigned as RBH if they are less than half the size of the longest sequence in the cluster. Our dataset contained a total of 6289 RBH clusters that were further used to reconstruct the phylogenetic tree. To do so, gene sequences were aligned using PRANK (https://pubmed.ncbi.nlm.nih.gov/24170401/) and GBLOCKS [87, 88] and a tree was built using IQTREE [89–92]. The tree obtained was then used to calculate corrected dN/dS using the method described in [76]. For dN/dS calculation we use all RBH clusters, aligned with PRANK (-t=guidetree.txt -f=phylips -prunetree -prunedata -translate -F -once -maxbranches = 0.15) using the appropriate pruned guide tree plus GBLOCKS (-t=c -b5=h ; data available in Dryad through the link: https://datadryad.org/stash/share/TieHWUYVZWsPHBeCkDkbQ-b_0B3NAsAFy6SUkD4ybrs.

dN and dS values for leafs were extracted using newick tools. Finally, we performed Wilcoxon tests on dN/dS values to compare the sequence evolution in sex-biased versus unbiased genes. To test for faster evolution of sex-biased genes along the *M. longipes* branch we performed a Generalized Linear Model (GLM) in pair-wise comparisons between *M. longipes* and its closest relative species (i.e. *M. pulchella* and *M. sp* (Cay)). To test the biological relevance of this result, we developed a bootstrap approach. On each iteration, we set all genes as unbiased and randomly assigned 253 genes as male sex-biased and 463 as female sex-biased. We then recalculated the differences in sequence evolution. We performed this bootstrap method for 1000 iterations and we defined the cut-off *p* value for 99.95% of samples for male-biased vs unbiased genes at 0.1631. This means that *p* values for male sex-biased genes are significant (0.0018) but also unlikely to have been obtained randomly (0.0018 < 0.1631).

### Sex-biased gene distribution between chromosomes

#### Sex chromosome identification

Gerromorphan karyotypes have previously been characterized as having either the XX/XY or XX/X0 sex determination systems [93, 94]. In *M. longipes*, Illumina genomic sequencing containing only males was used to align genomic reads against *M. longipes* genome and extract the genomic coverage of each scaffold. The scaffold 1893 was the only scaffold among the 13 biggest scaffolds (more than 90% of the genome) that presented twice less coverage than the other scaffolds. To finally assess the identity of the X chromosome in *M. longipes*, we monitored the gene expression and found that the scaffold 1893 included both male- and female-biased genes, excluding this scaffold to be the Y chromosome. We also looked for a possible Y chromosome by identifying scaffolds with similar genomic coverage as the X chromosome but containing genes with only male-biased expression. We did not find any among the fifty largest scaffolds, suggesting either that *M. longipes* has a XX/X0 sex determination system or that our genome assembly presents a highly fragmented Y chromosome.

#### Genomic distribution of sex-biased genes

We identified the genomic location of each gene and selected genes with a fold change superior to 1.5 between males and females as sex-biased genes (Padj < 0.05). Over- or under-representation of sex-biased genes in the X chromosome (scaffold 1893) compared to the autosomes (12 other largest scaffolds) was tested using Fisher’s exact tests.

#### Estimation of dosage compensation

To compare the average level of gene expression between males and females in the X chromosome we first selected expressed genes with FPKM > 2 in at least half of the samples (12 samples per leg). We also averaged gene expressions between replicates and lines before testing for differences in expression (Wilcoxon tests on the log2 (FPKM)).

### Detection of large sex-biased gene regions

To detect large chromosomal regions enriched in sex-biased genes we developed a bootstrapping method based on sliding windows of 2 Mb with a step size of 100 kb (Additional file 17: Figure S10). Gene density calculation revealed that on average, genes are found every 20 kb in *M. longipes* genome. This pattern was homogeneous among chromosomes (Additional file 18: Table S8). We therefore split each chromosome into bins of 100 kb and generated sliding windows of 2 Mb (20 bins) to include approximately 100 genes per window in the analysis (Additional file 17: Figure S10B, Additional file 18: Table S8). We used two scaffolds, one scaffold with two enriched regions (scaffold 2) and a scaffold with no enriched region (scaffold 1914), to repeat the analysis with smaller regions (1 Mb, 500 kb, 250 kb, and 120 kb). We found similar results in both scaffolds, regardless of the size of the region, indicating that our analysis is statistically robust and is not missing information.

#### Fold-change reassignment and gene position

From the DESeq2 analyses, all expressed genes were associated with a log2 fold change (Log2FC) and a *p* value (Padj). Unexpressed genes (FPKM < 2) were assigned a log2FC of 0 and a *p* value of 1. Among the expressed genes, we switched the log2FC to 0 for the unbiased genes (Padj > 0.05), in order to directly assess sex-biased genes based on log2FC values (Additional file 17: Figure 10A).

In a second step, we merged the dataset on sex-biased expression with the gene positions (Additional file 17: Figure S10A).

#### Genome-wide detection of sex-biased gene regions

A mean log2FC was calculated for each window and reported along the chromosomes to reveal genome-wide regions of sex-biased genes (Additional file 17: Figure 10B).

#### Bootstrapping method

To test whether these regions are significantly enriched in male or female-biased genes, we developed a bootstrap approach (Additional file 17: Figure S10C). As the mean expression level of a gene influences the log2FC value (i.e., genes with low expressions are more likely to have high log2FC values and genes with high expression are more likely to be differentially expressed), we created 5 categories of genes based on their expression levels (baseMean values from DESeq2 tables; Additional file 15: Table S7). We then reassigned randomly, within each category, the log2FC at each gene position in the genome. This step was performed 100,000 times, therefore generating 100,000 random log2FC profiles.

Finally, to test for the significant enrichment of gene in these regions, we compared for each bin the observed log2FC values with the log2FC values generated from the bootstrap. To call for significantly enriched region of sex-biased genes, we identified regions for which the observed log2FC value was higher (male-biased) or lower (female-biased) than expected randomly after applying a Bonferroni correction (Additional file 17: Figure S10D), correcting the bootstrap values by the total number of independent windows in the genome (*n* = 300). We then applied a cut-off value of 99.99% to define significantly enriched regions. We note that this analysis is rather stringent (possibly missing other important enriched regions), but the highlighted regions are unambiguous (low sensitivity but high robustness).

### Detection of clusters of consecutive sex-biased genes

This analysis was primarily inspired from Boutanaev et al. [11]. In short, we determined clusters by ordering genes along the genome and detecting regions of consecutive male- or female-biased genes (Padj < 0.05). To avoid identifying clusters overlapping two different chromosomes, we performed this analysis on the thirteen largest scaffolds separately. We then tested whether the observed distribution of genes differed from a stochastic distribution by randomly assigning a genomic position to unbiased, male-biased, and female-biased genes, respectively. The proportion of sex-biased genes found in clusters as well as the distribution of cluster sizes was calculated by averaging 1000 iterations (Additional file 17: Figure S10). *P* values were extracted from the 95% fluctuation intervals calculated from the 1000 randomized iterations.

### Gene ontology analysis

Gene names and functions were annotated by sequence similarity against the NCBI “non redundant” protein database using Blast2GO. The Blast2GO annotation was then provided to detect Gene Ontology terms enrichment (*p* value < 0.05) using the default method of TopGO R package version 2.34.0.

## Supplementary Information

**Additional file 1: Table S1.** Genome metrics after assembly and genomic libraries used to sequence and assemble *M. longipes* genome.

**Additional file 2: Figure S1.** Diagram of BUSCO analysis.

**Additional file 3: Figure S2.** Experimental design of the comparative transcriptomic analysis.

**Additional file 4: Table S2.** Summary statistics of the differences in fold-change and expression for the sex-biased genes between the three legs.

**Additional file 5: Figure S3.** Comparison of gene expression (log2FPKM + 1) across legs for male- and female-biased genes identified in each leg respectively.

**Additional file 6: Table S3.** Fisher test statistical analyses of leg and sex biased genes.

**Additional file 7: Figure S4.** Crosstalk between leg- and sex-biased genes.

**Additional file 8: Table S4.** Summary statistics for dN/dS analyses.

**Additional file 9: Figure S5.** Interaction plots on dNdS medians on the six *Microvelia* species.

**Additional file 10: Figure S6.** Gene expression correlation between male and female transcriptomes for each gene on the X chromosome.

**Additional file 11: Figure S7.** Genome-wide characterization of large genomic clusters.

**Additional file 12: Figure S8.** Distributions of the proportion of clustered sex-biased genes.

**Additional file 13: Table S5.** Gene Ontology (GO) term tables.

**Additional file 14: Table S6.** Transcriptome metrics.

**Additional file 15: Table S7.** Summarizing tables on raw read counts, FPKM counts, blast analysis and the lists of differentially expressed (DE) genes between males and females across legs.

**Additional file 16: Figure 9.** Venn diagrams on sex-biased genes identified on the two lines.

**Additional file 17: Figure S10.** General pipeline of the bootstrap analysis to detect large genomic clusters of sex-biased genes.

**Additional file 18: Table S8.** Summary table of the average gene number and density per scaffold.

## Data Availability

All data generated or analyzed during this study are included in this published article, its supplementary information files, and publicly available repositories. - BioProject ID: PRJNA610161 and PRJNA642372. - Dryad link to dN/dS data analyses and scripts employed in this study: https://datadryad.org/stash/share/TieHWUYVZWsPHBeCkDkbQ-b_0B3NAsAFy6SUkD4ybrs.
