# Supplementary material for: Impact of male trait exaggeration on sex-biased gene expression and genome architecture in a water strider
Source: BMC Biol. 2021 Apr 30;19:89. doi: 10.1186/s12915-021-01021-4 (PMC8088084; doi:10.1186/s12915-021-01021-4)
Supplement: Supplementary file 2 — Additional file 2: Figure S1. Diagram of BUSCO analysis. [file 12915_2021_1021_MOESM2_ESM.docx]

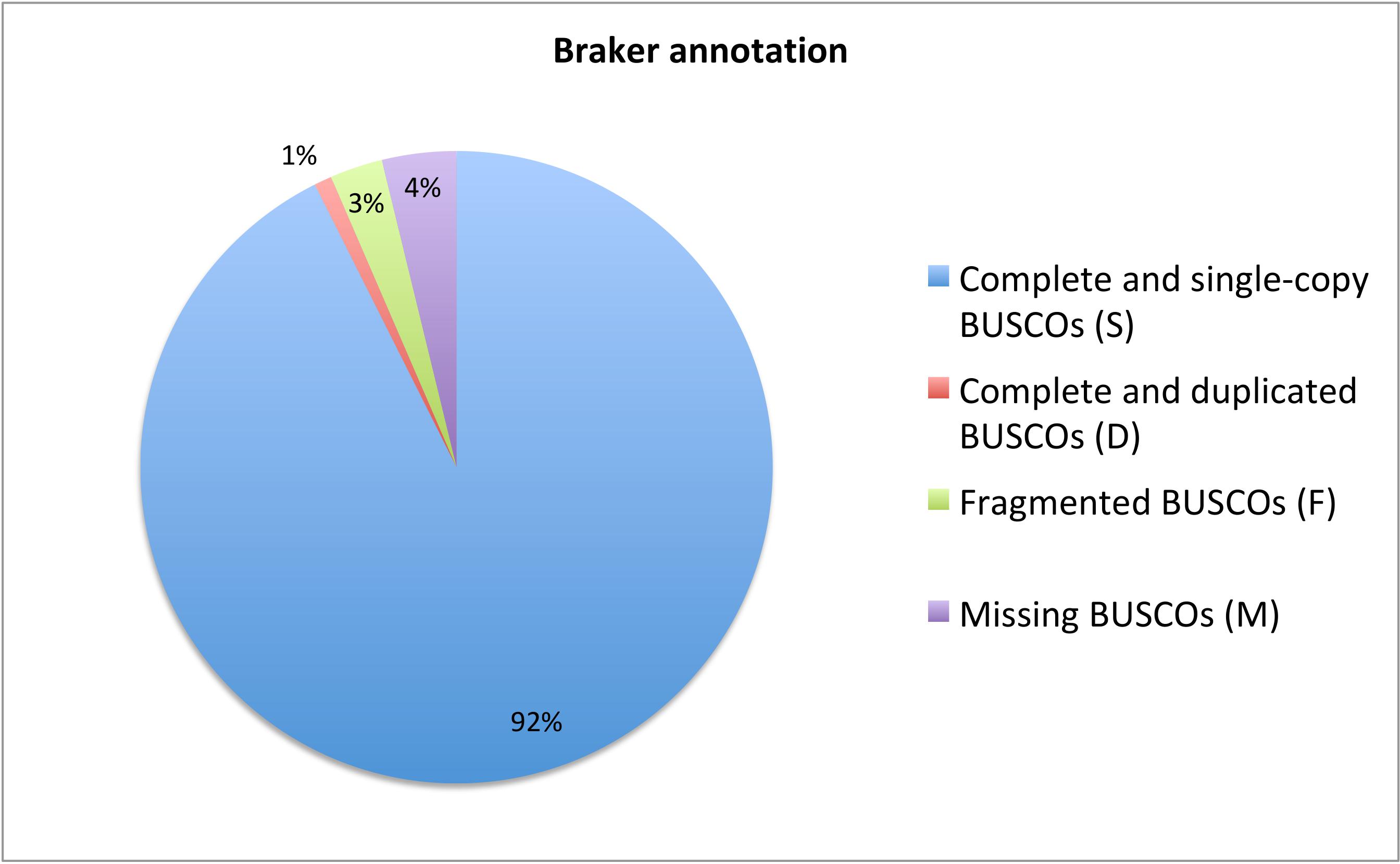


**Additional file 2: Figure S1:** Diagram of BUSCO analysis run for the 26 130 genes identified from Braker annotation.
