# Supplementary material for: Impact of male trait exaggeration on sex-biased gene expression and genome architecture in a water strider
Source: BMC Biol. 2021 Apr 30;19:89. doi: 10.1186/s12915-021-01021-4 (PMC8088084; doi:10.1186/s12915-021-01021-4)
Supplement: Supplementary file 3 — Additional file 3: Figure S2. Experimental design of the comparative transcriptomic analysis. [file 12915_2021_1021_MOESM3_ESM.docx]

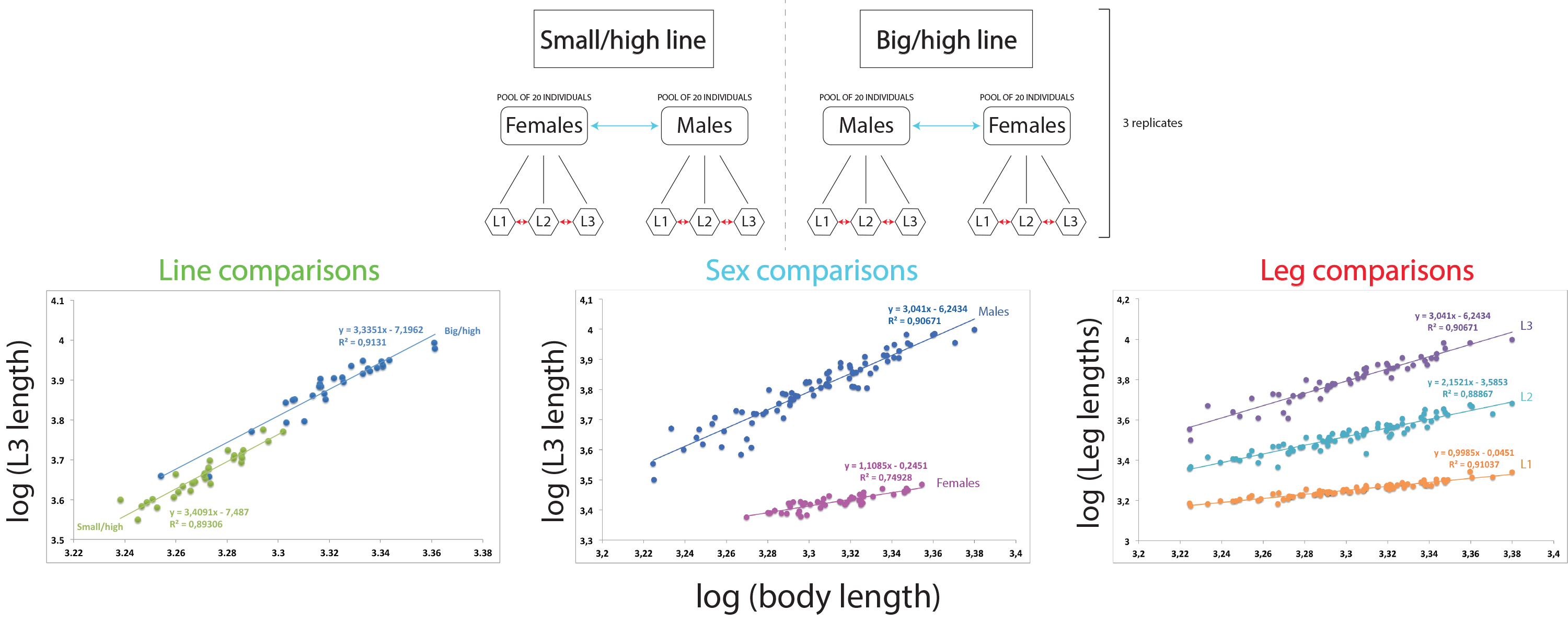


**Additional file 3: Figure S2:** Experimental design of the comparative transcriptomic analysis. Top panel represents the three different conditions used in the transcriptomic analysis, namely the line, sex and leg conditions. The comparative transcriptomics was performed on three replicates and a pool of 20 individuals per replicate. Bottom panel indicates allometric differences between all three conditions.
