# Supplementary material for: Impact of male trait exaggeration on sex-biased gene expression and genome architecture in a water strider
Source: BMC Biol. 2021 Apr 30;19:89. doi: 10.1186/s12915-021-01021-4 (PMC8088084; doi:10.1186/s12915-021-01021-4)
Supplement: Supplementary file 5 — Additional file 5: Figure S3. Comparison of gene expression (log2FPKM + 1) across legs for male- and female-biased genes identified in each leg respectively. [file 12915_2021_1021_MOESM5_ESM.docx]

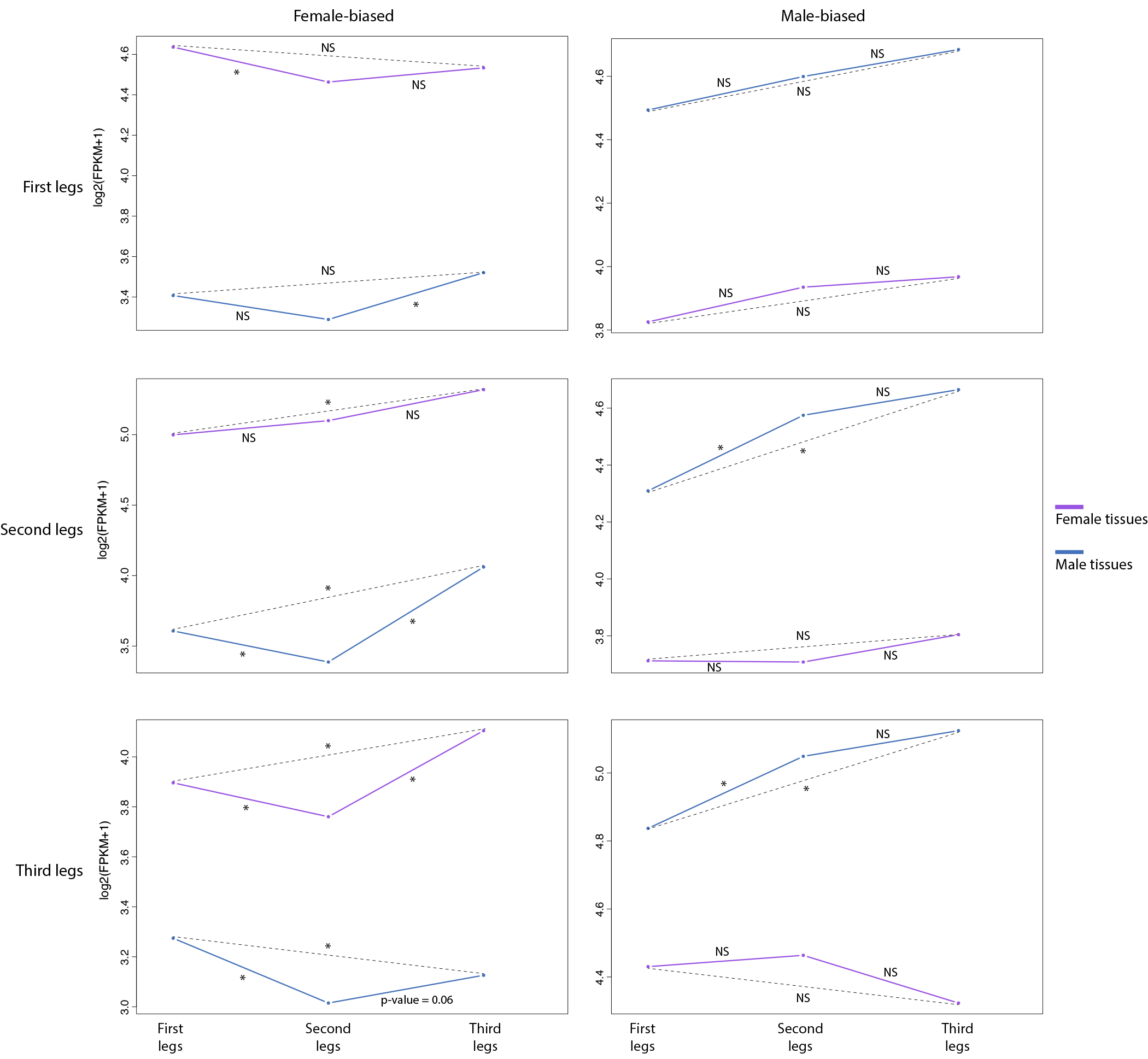


**Additional file 5: Figure S3:** Comparison of gene expression (log2FPKM+1) across legs for male- and female-biased genes identified in each leg respectively.
