# Supplementary material for: Impact of male trait exaggeration on sex-biased gene expression and genome architecture in a water strider
Source: BMC Biol. 2021 Apr 30;19:89. doi: 10.1186/s12915-021-01021-4 (PMC8088084; doi:10.1186/s12915-021-01021-4)
Supplement: Supplementary file 7 — Additional file 7: Figure S4. Crosstalk between leg- and sex-biased genes. [file 12915_2021_1021_MOESM7_ESM.docx]

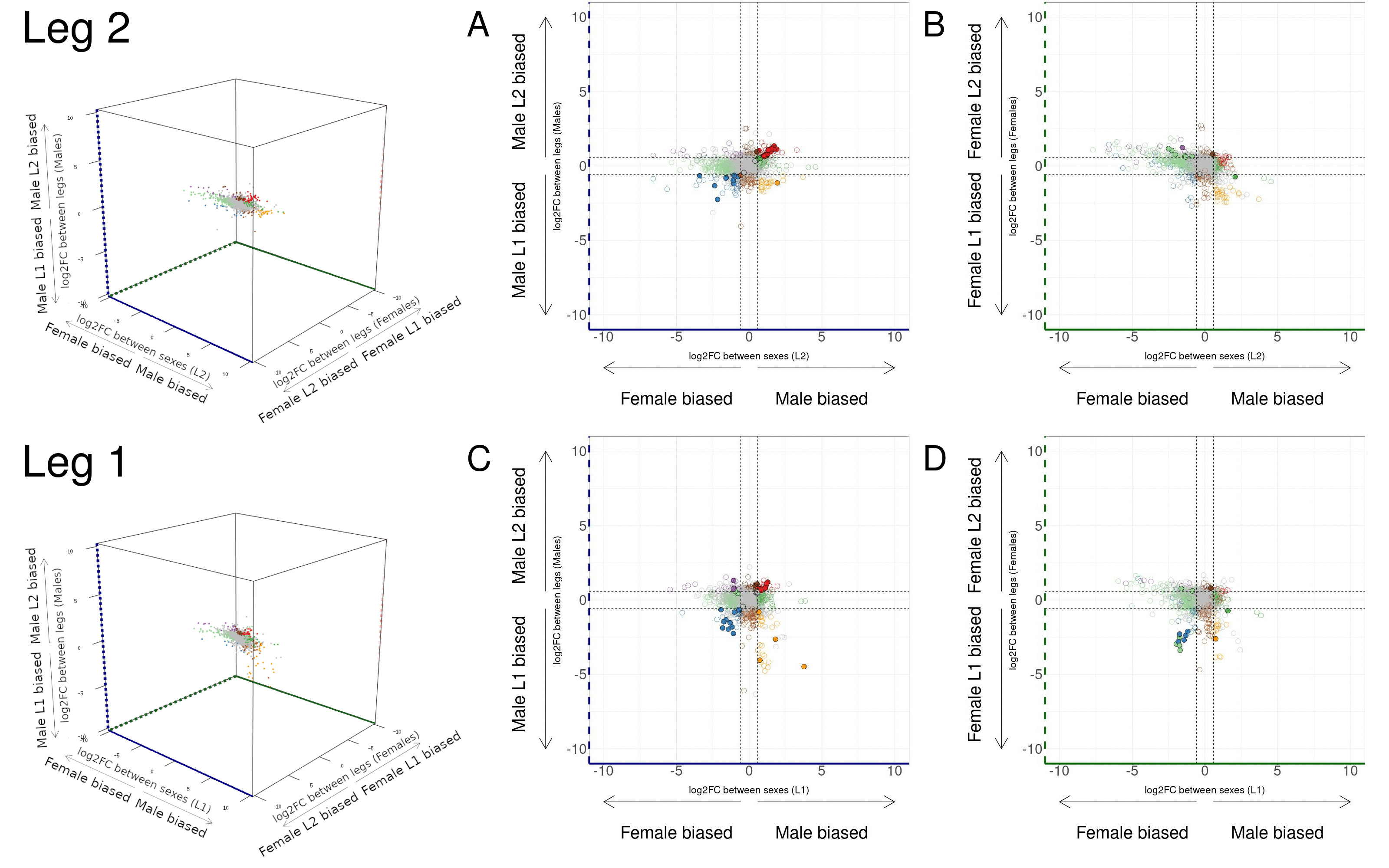


**Additional file 7: Figure S4:** Crosstalk between leg- and sex-biased genes. (**A**) Comparison between sex-biased genes in the second legs and leg-biased genes in males. (**B**) Comparison between sex-biased genes in the second legs and leg-biased genes in females. (**C**) Comparison between sex-biased genes in the first legs and leg-biased genes in males. (**D**) Comparison between sex-biased genes in the first legs and leg-biased genes in females. Colour code in A and B represents the same genes in these two panels, and colour code in C and D represents the same genes in these two panels. Gens are based on sex biased and leg biased expression in males (log2FC>log2(1.5)) : Purple=Female-biased & Leg 2 biased; Dark brown=Sex unbiased & Leg 2 biased; Red=Male-biased & Leg 2 biased; Light green=Female-biased & Leg unbiased; Grey=Sex unbiased & Leg unbiased; Dark green=Male-biased & Leg unbiased; Blue=Female-biased & Leg 1 biased; Light brown=Sex unbiased & Leg 1 biased; Orange=Male-biased & Leg 1 biased. Filled circles indicate genes with padj<0.05 in both conditions (sex- and leg-biased). Hollow circles indicate genes with padj>0.05 in one or both conditions.
