# Supplementary figures and images for: Impact of male trait exaggeration on sex-biased gene expression and genome architecture in a water strider
Source: BMC Biol. 2021 Apr 30;19:89. doi: 10.1186/s12915-021-01021-4 (PMC8088084; doi:10.1186/s12915-021-01021-4)

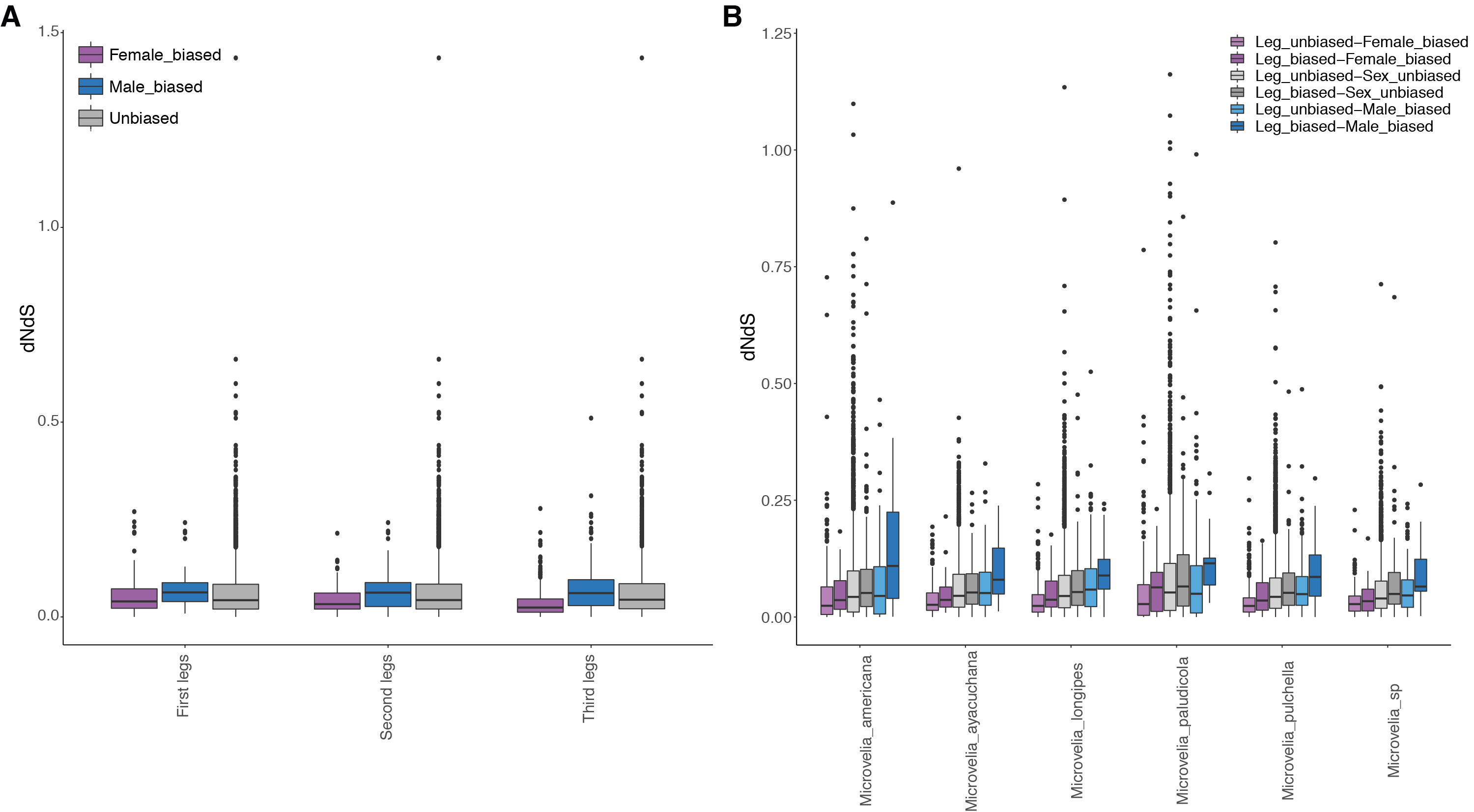


**Additional file 9: Figure S5:** Interaction plots on dNdS medians on the six *Microvelia* species.

Supplement: Supplementary file 9 — Additional file 9: Figure S5. Interaction plots on dNdS medians on the six Microvelia species. [file 12915_2021_1021_MOESM9_ESM.docx]

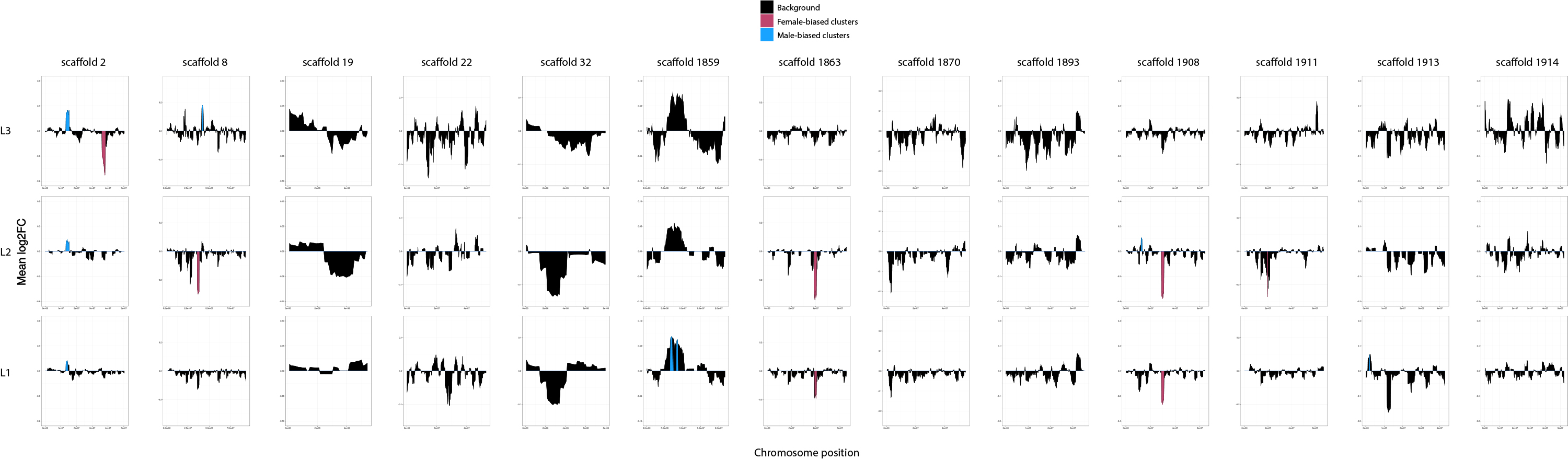


**Additional file 11: Figure S7:** Genome-wide characterization of large genomic clusters.

Supplement: Supplementary file 11 — Additional file 11: Figure S7. Genome-wide characterization of large genomic clusters. [file 12915_2021_1021_MOESM11_ESM.docx]

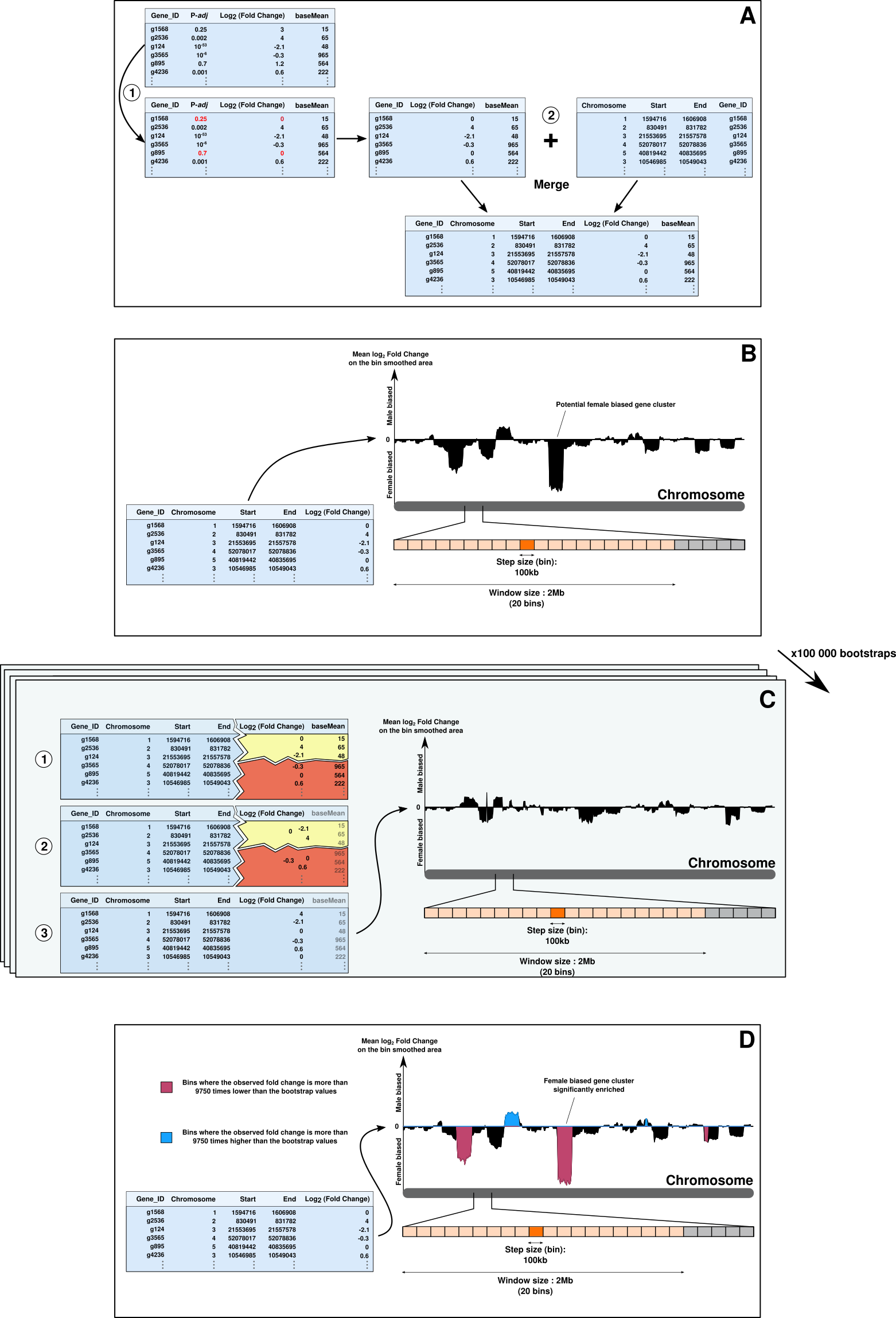

Supplement: Supplementary file 17 — Additional file 17: Figure S10. General pipeline of the bootstrap analysis to detect large genomic clusters of sex-biased genes. [file 12915_2021_1021_MOESM17_ESM.png]
