# Supplementary material for: Impact of male trait exaggeration on sex-biased gene expression and genome architecture in a water strider
Source: BMC Biol. 2021 Apr 30;19:89. doi: 10.1186/s12915-021-01021-4 (PMC8088084; doi:10.1186/s12915-021-01021-4)
Supplement: Supplementary file 10 — Additional file 10: Figure S6. Gene expression correlation between male and female transcriptomes for each gene on the X chromosome. [file 12915_2021_1021_MOESM10_ESM.docx]

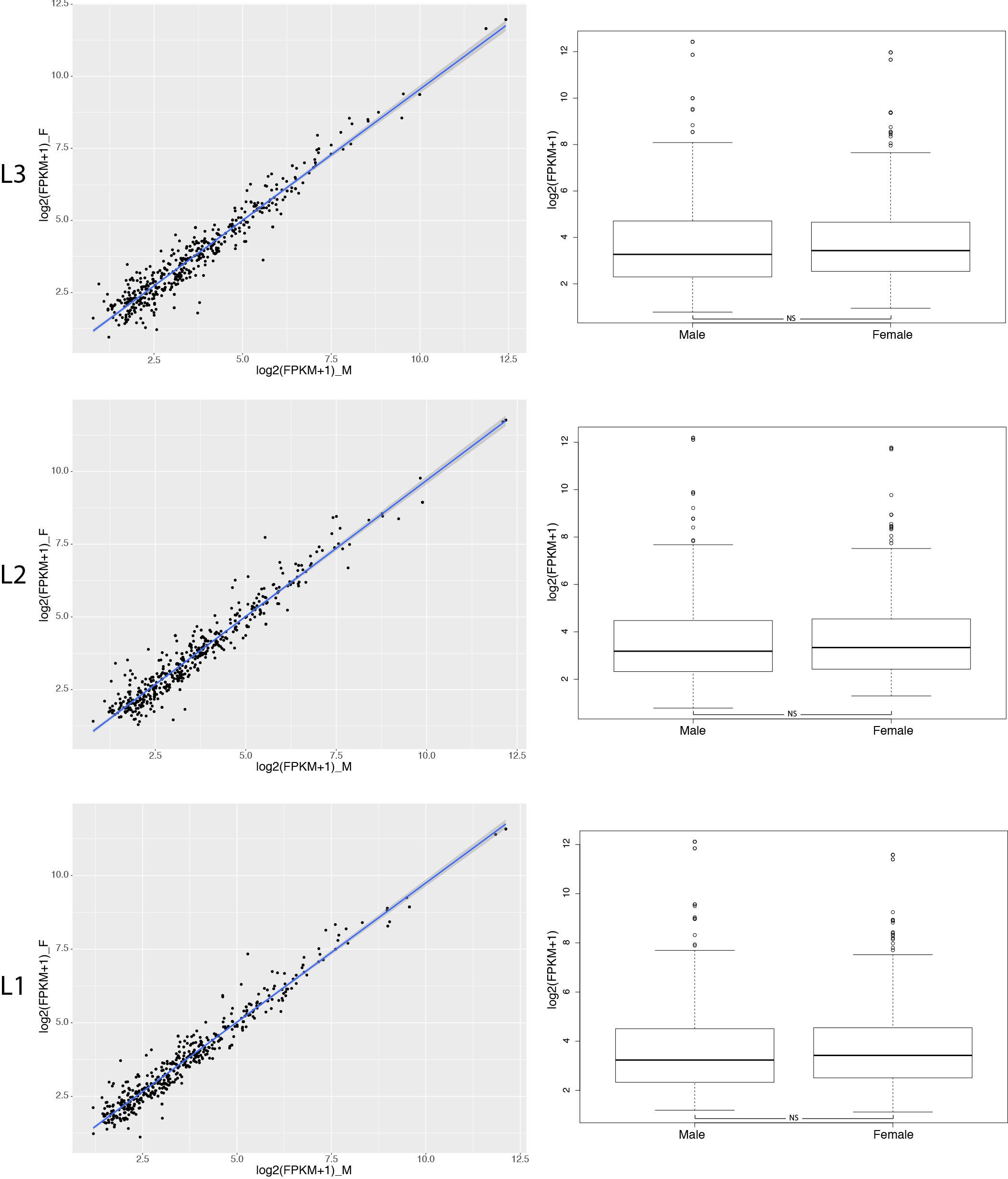


**Additional file 10: Figure S6:** Left panels show gene expression correlation between male and female transcriptomes for each gene on the X chromosome. The analysis was performed for the three different legs and regressions were fitted from a linear model. Right panels indicate average gene expression differences between males and females on the X chromosome for the three legs.
