# Supplementary material for: Impact of male trait exaggeration on sex-biased gene expression and genome architecture in a water strider
Source: BMC Biol. 2021 Apr 30;19:89. doi: 10.1186/s12915-021-01021-4 (PMC8088084; doi:10.1186/s12915-021-01021-4)
Supplement: Supplementary file 12 — Additional file 12: Figure S8. Distributions of the proportion of clustered sex-biased genes. [file 12915_2021_1021_MOESM12_ESM.docx]

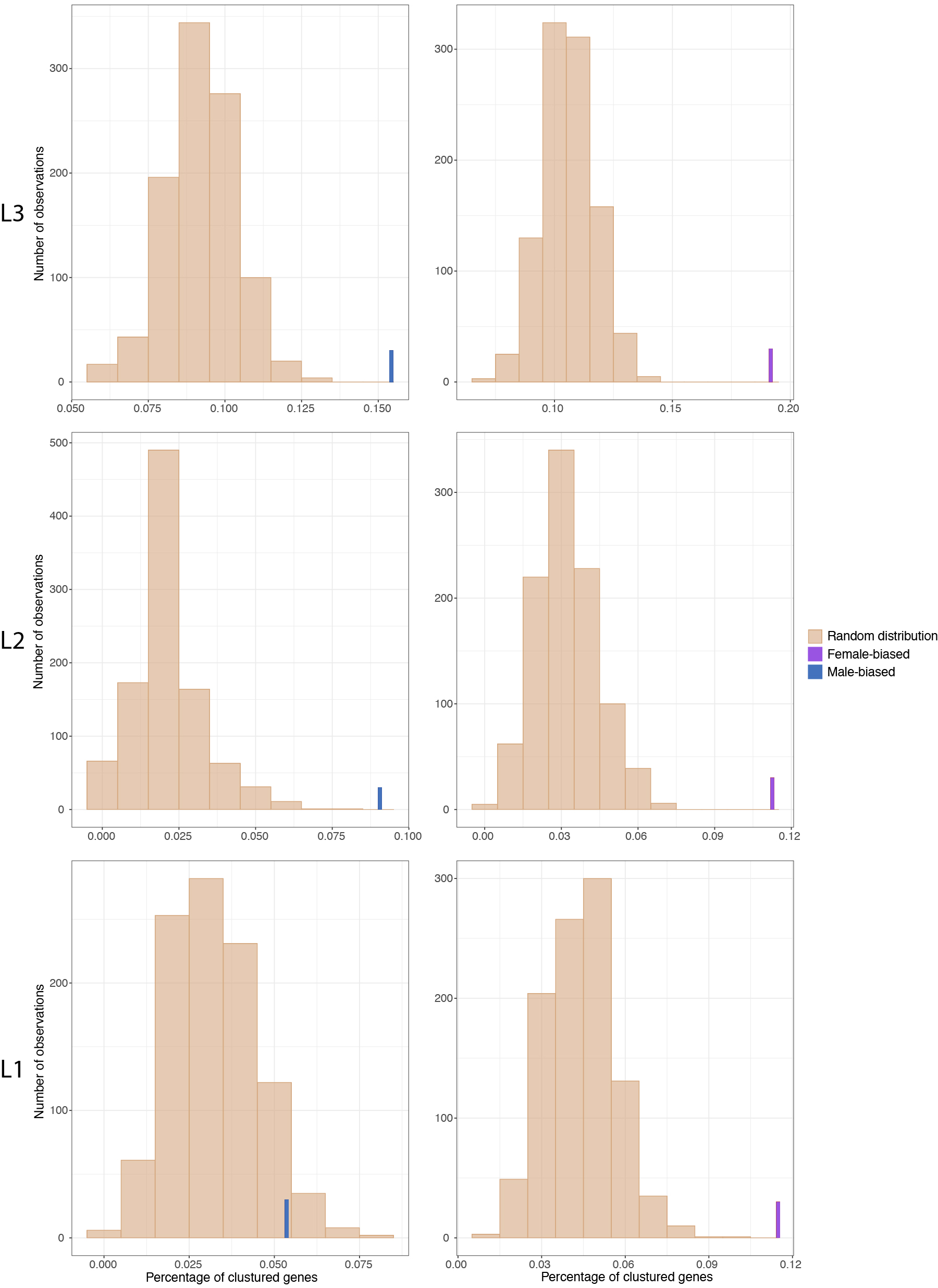


**Additional file 12: Figure S8:** Distributions of the proportion of clustered sex-biased genes (at least two consecutive sex-biased genes) generated through 1000 random iterations in each leg separately. Blue and purple bars correspond to the observed proportions of male- and female-biased genes, respectively, in the three legs.
