# Supplementary material for: Impact of male trait exaggeration on sex-biased gene expression and genome architecture in a water strider
Source: BMC Biol. 2021 Apr 30;19:89. doi: 10.1186/s12915-021-01021-4 (PMC8088084; doi:10.1186/s12915-021-01021-4)
Supplement: Supplementary file 16 — Additional file 16: Figure 9. Venn diagrams on sex-biased genes identified on the two lines. [file 12915_2021_1021_MOESM16_ESM.docx]

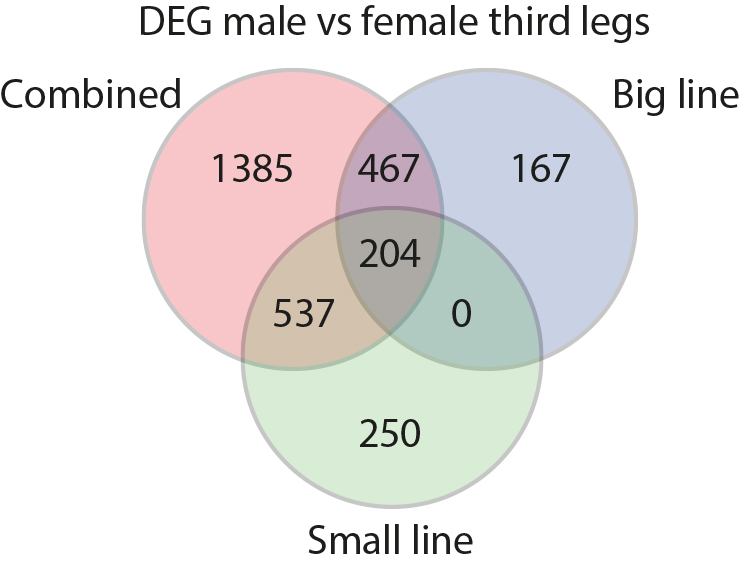


**Additional file 16: Figure 9:** Venn diagrams on sex-biased genes identified on the two lines separately or combined (see material and methods for more details).
